# Supplementary material for: An electronic origin of charge order in infinite-layer nickelates
Source: Nat Commun. 2023 Sep 6;14:5477. doi: 10.1038/s41467-023-41236-3 (PMC10482875; doi:10.1038/s41467-023-41236-3)
Supplement: Supplementary file 1 — Supplementary Information [file 41467_2023_41236_MOESM1_ESM.pdf]

# Supplementary Information of "An electronic origin of charge order in infinite-layer nickelates"


Hanghui Chen,[1,2] Yi-feng Yang,[3,4,5] Guang-Ming Zhang,[6,7] and Hongquan Liu[1]

[1]*NYU-ECNU Institute of Physics, NYU Shanghai, Shanghai 200122, China*

[2]*Department of Physics, New York University, New York, New York 10012, USA*

[3]*Beijing National Laboratory for Condensed Matter Physics and Institute of Physics, Chinese Academy of Sciences, Beijing 100190, China*

[4]*University of Chinese Academy of Sciences, Beijing 100190, China*

[5]*Songshan Lake Materials Laboratory, Dongguan, Guangdong 523808, China*

[6]*State Key Laboratory of Low-Dimensional Quantum Physics and Department of Physics, Tsinghua University, Beijing 100084, China*

[7]*Frontier Science Center for Quantum Information, Beijing 100084, China*



(Dated: August 13, 2023)

## Contents

Supplementary Note 1. The 17-orbital model 3

Supplementary Note 2. Fitting to the DFT band structure 4

Supplementary Note 3. Hubbard interaction on Nd-$d$ orbitals in the 17-orbital model 6

Supplementary Note 4. The Ni effective mass at different $U_d$ values 8

Supplementary Note 5. Possible structural distortions in the CO state 10

Supplementary Note 6. Comparison of the PM and CO spectral functions using the low-energy effective model 12

Supplementary Note 7. Total energy as a function of $E_{ds}$ calculated with $U_d = 4$ eV 13

Supplementary Note 8. Comparison of competing states using the low-energy effective model 14

References 15

## Supplementary Note 1. THE 17-ORBITAL MODEL

In this section, we provide the explicit expression of the interacting 17-orbital model. The full Hamiltonian reads:

$$\hat{H} = \hat{H}_0 + \hat{H}_{\text{int}} - \hat{V}^{\text{dc}} \tag{1}$$

The non-interacting part reads:

$$\hat{H}_0 = \sum_{\mathbf{k},mm',\sigma} \mathcal{H}_0(\mathbf{k})_{mm'} \hat{c}^\dagger_{\mathbf{k}m\sigma} \hat{c}_{\mathbf{k}m'\sigma} \tag{2}$$

where $\hat{c}^\dagger_{\mathbf{k}m\sigma}$ is the creation operator with crystal momentum $\mathbf{k}$, orbital $m$ and spin $\sigma$. $\mathcal{H}_0(\mathbf{k})$ is constructed by the maximally localized Wannier function (MLWF):

$$\mathcal{H}_0(\mathbf{k}) = \sum_{\mathbf{R}} \exp(i\mathbf{k}\cdot\mathbf{R})\mathcal{H}_0(\mathbf{R}) \tag{3}$$

where $\mathcal{H}_0(\mathbf{R})$ is the non-interacting Hamiltonian in the Wannier basis [1].

The interaction is of Slater-Kanamori type [2]:

$$\begin{aligned}\hat{H}_{\text{int}} = U_{\text{Ni}} \sum_{i,m} \hat{n}_{im\uparrow}\hat{n}_{im\downarrow} + \sum_{i,m>m',\sigma\sigma'} \left[ (U_{\text{Ni}} - 2J_{\text{Ni}} - \delta_{\sigma\sigma'})\hat{n}_{im\sigma}\hat{n}_{im'\sigma'} \right] \\ - \sum_{i,m\neq m'} J_{\text{Ni}} \left( \hat{c}^\dagger_{im\downarrow}\hat{c}^\dagger_{im'\uparrow}\hat{c}_{im'\downarrow}\hat{c}_{im\uparrow} + \hat{c}^\dagger_{im'\uparrow}\hat{c}^\dagger_{im'\downarrow}\hat{c}_{im\uparrow}\hat{c}_{im\downarrow} + \text{h.c.} \right)\end{aligned} \tag{4}$$

where $\hat{c}^\dagger_{im\sigma}$ is the creation operator with site $i$, orbital $m$ and spin $\sigma$. $\hat{n}_{im\sigma} = \hat{c}^\dagger_{im\sigma}\hat{c}_{im\sigma}$ is the occupancy operator. In the 17-orbital model, the MLWF for the Ni-$d$ orbitals are very localized and atomic-like (see Table I), we use a Hubbard $U_{\text{Ni}} = 10$ eV and a Hund's exchange $J_{\text{Ni}} = 1$ eV, which has proved to be reasonable for this large energy window treatment of transition metal oxides [3–6]. The dependence of the main results on $U_{\text{Ni}}$ and $U_{\text{Ni}}$ is discussed in the main text.

Considering that nominally Ni ions have a $d^9$ occupancy in $NdNiO_2$, we employ the fully-localized-limit (FLL) double counting [7], following the previous works [8–16]. The double counting potential reads:

$$V^{\text{dc}} = U_{\text{Ni}}\left(N_d - \frac{1}{2}\right) - J_{\text{Ni}}\left(\frac{5}{2}N_d - \frac{5}{2}\right) \tag{5}$$

where $N_d$ is the occupancy of Ni-$d$ orbitals.

## Supplementary Note 2. FITTING TO THE DFT BAND STRUCTURE

In this section we show the band structure computed from the non-interacting part of different models and compare it to the DFT-calculated band structure of $NdNiO_2$. Panel (a) of Fig. 1 shows the band structure computed from the 17-orbital model (red dots) with all the hopping parameters determined by the maximally localized Wannier functions [17], while the blue curves are the DFT-calculated band structure of $NdNiO_2$. The spread of each Wannier function is shown in Table I. Panel (b) of Fig. 1 shows the band structure computed from the low-energy effective model Eq. (1) in the main text (red dots) with the parameters shown in Table I in the Methods, while the blue curves are the DFT-calculated band structure of $NdNiO_2$. The 17-orbital model well reproduces the DFT band structure of $NdNiO_2$ in an energy window about 15 eV around the Fermi level. The low-energy effective model reproduces the two bands of $NdNiO_2$ that cross the Fermi level.

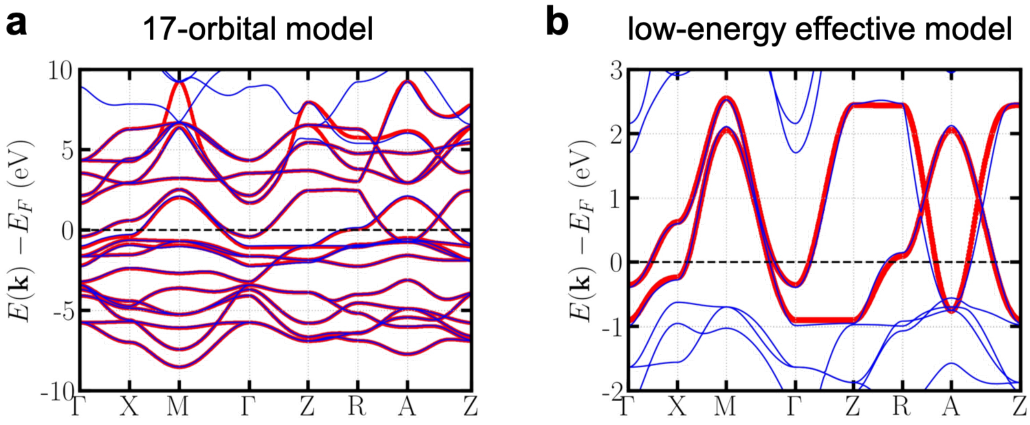


Supplementary Figure 1: (a) The band structure computed from the 17-orbital tight-binding Hamiltonian (red dots) is compared to the DFT-calculated band structure (blue curves). (b) The band structure computed from the non-interacting part of the low-energy effective model (red dots) is compared to the DFT-calculated band structure (blue curves). In both panels, the black dashed line highlights the Fermi level and the coordinates of the high-symmetry **k**-points are: Γ(0, 0, 0), X(0.5, 0, 0), M(0.5, 0.5, 0), Z(0, 0, 0.5), R(0.5, 0, 0.5), A(0.5, 0.5, 0.5).

Supplementary Table I: Spread of Wannier functions in the 17-orbital model, which includes five Ni-$d$ orbitals, five Nd-$d$ orbitals, six O-$p$ orbitals and one interstitial-$s$ orbital per primitive cell. The spread has the unit of Å$^2$.

| | | | | | | | |
|---|---|---|---|---|---|---|---|
| orbital | Ni-$d_{3z^2-r^2}$ | Ni-$d_{xz}$ | Ni-$d_{yz}$ | Ni-$d_{x^2-y^2}$ | Ni-$d_{xy}$ | – | – |
| spread | 0.51 | 0.47 | 0.47 | 0.45 | 0.45 | – | – |
| orbital | Nd-$d_{3z^2-r^2}$ | Nd-$d_{xz}$ | Nd-$d_{yz}$ | Nd-$d_{x^2-y^2}$ | Nd-$d_{xy}$ | – | – |
| spread | 2.51 | 2.14 | 2.14 | 2.64 | 2.60 | – | – |
| orbital | O1-$p_z$ | O1-$p_x$ | O1-$p_y$ | O2-$p_z$ | O2-$p_x$ | O2-$p_y$ | $s$ |
| spread | 0.79 | 0.84 | 0.93 | 0.79 | 0.93 | 0.84 | 2.78 |

## Supplementary Note 3. HUBBARD INTERACTION ON Nd-$d$ ORBITALS IN THE 17-ORBITAL MODEL

In the 17-orbital model, the spread of the MLWF for Nd-$d$ orbitals is substantially larger than the MLWF for Ni-$d$ orbitals. This means that the Hubbard $U$ interaction should be weaker on Nd-$d$ orbitals [18]. We test the correlation effects from Nd-$d$ orbitals by turning on an additional Hubbard interaction of Slater-Kanamori form with $U_{\mathrm{Nd}} = 4$ eV and $J_{\mathrm{Nd}} = 0.4$ eV in the 17-orbital model. We re-calculate its spectral function and show the result in Fig. 2. The result does not qualitatively change as long as $U_{\mathrm{Nd}}$ and $J_{\mathrm{Nd}}$ are of reasonable values. We find that considering the Hubbard interaction on Nd-$d$ orbitals does not affect the charge-disproportionation on Ni-$d$ orbital nor the charge transfer from Ni-$d$ to Nd-$d$/interstitial-$s$ orbitals. The new spectral functions are very similar to those shown in Fig. 1 in the main text.

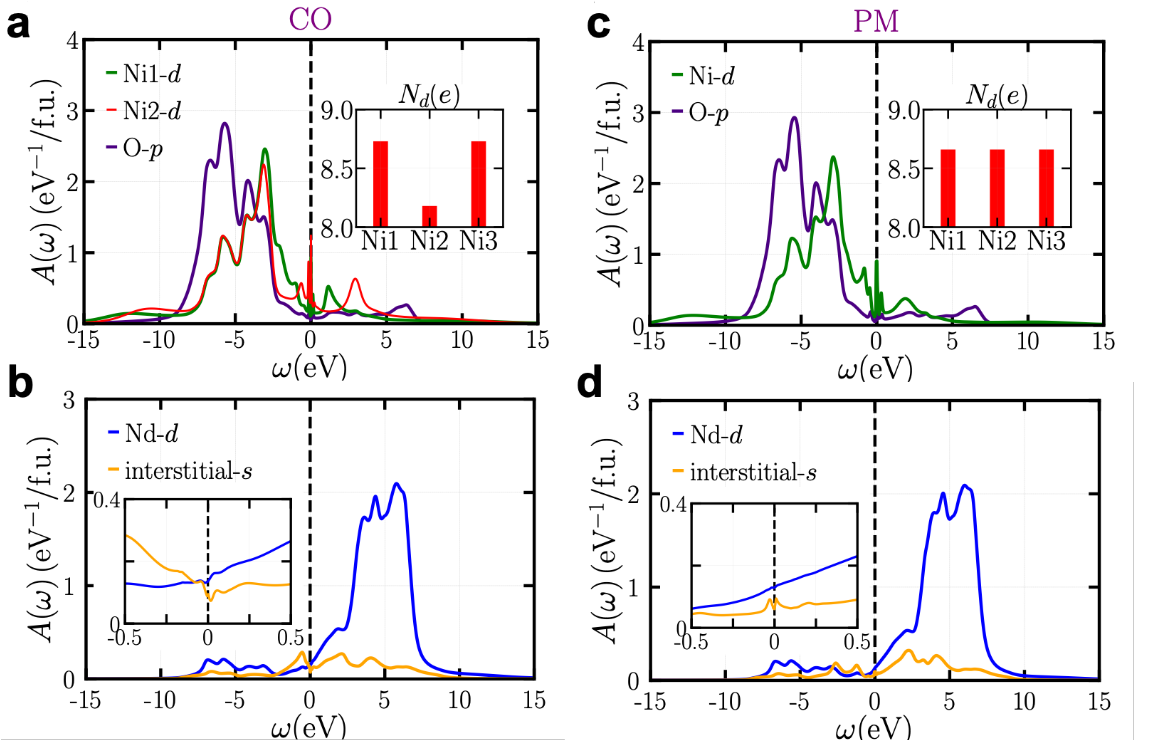


Supplementary Figure 2: Spectral functions of $NdNiO_2$ calculated by DFT+DMFT method using the 17-orbital model with $U_{\mathrm{Ni}} = 10$ eV, $J_{\mathrm{Ni}} = 1$ eV and $U_{\mathrm{Nd}} = 4$ eV, $J_{\mathrm{Nd}} = 0.4$ eV. (a) and (b): The charge ordered state (CO). (a): The green, red and purple curves are Ni1-$d$, Ni2-$d$ and O-$p$ projected spectral functions, respectively. The inset shows the site-resolved Ni-$d$ occupancy. The $d$ occupancy of Ni2 site is substantially smaller than that of Ni1 and Ni3 sites. (b): The blue and orange curves are Nd-$d$ and interstitial-$s$ projected spectral functions, respectively. The inset shows the energy range near the Fermi level. (c) and (d): The uniform paramagnetic state (PM). (c): The green and purple curves are Ni-$d$ and O-$p$ projected spectral functions, respectively. The inset shows the site-resolved Ni-$d$ occupancy. (d): identical to (b) but for the PM state. In all panels, the dashed line is the Fermi level, which is shifted to zero point.

## Supplementary Note 4. THE Ni EFFECTIVE MASS AT DIFFERENT $U_d$ VALUES

In this section, we study the effect mass of Ni-$d_{x^2-y^2}$ orbital in the low-energy effective model (Eq. (1) in the main text). Since the state is metallic, in local DMFT calculations, we can directly relate the effective mass to the quasi-particle weight $Z$, which is defined from the real part of the retarded self-energy on the real-frequency axis:

$$\frac{m^*}{m} = \frac{1}{Z} = 1 - \frac{\partial \mathrm{Re}\Sigma(\omega + i0^+)}{\partial \omega}\Big|_{\omega=0} \tag{6}$$

However, the self-energy from CTQMC is calculated on the Matsubara frequencies. If the low-frequency properties are reasonably well described by the Fermi-liquid fixed point, the low-frequency limit of the real-frequency self-energy may be inferred with reasonable accuracy from the data at small Matsubara frequencies [19]:

$$\frac{m^*}{m} = \frac{1}{Z} \simeq 1 - \frac{d\mathrm{Im}\Sigma(i\omega_n)}{d\omega_n}\Big|_{\omega_n\to 0} \tag{7}$$

In practice, we extract the effective mass by fitting a fourth-order polynomial to the first six Matsubara-axis data points for $\mathrm{Im}\Sigma(i\omega_n)$ and computing the needed quantities from the fitting function. Fig. 3 shows $\mathrm{Im}\Sigma(i\omega_n)$ calculated by using the low-energy effective model with $U_d = 3.7$ eV. Panel (a) shows a large frequency range and panel (b) shows the self-energy of the first six Matsubara frequencies. From the fitting, we obtain that at $U_d = 3.7$ eV, the effective mass of Ni-$d_{x^2-y^2}$ orbital $m^*/m$ is 5.5, which is consistent with the result from the full *ab initio* GW+EDMFT calculations [20].

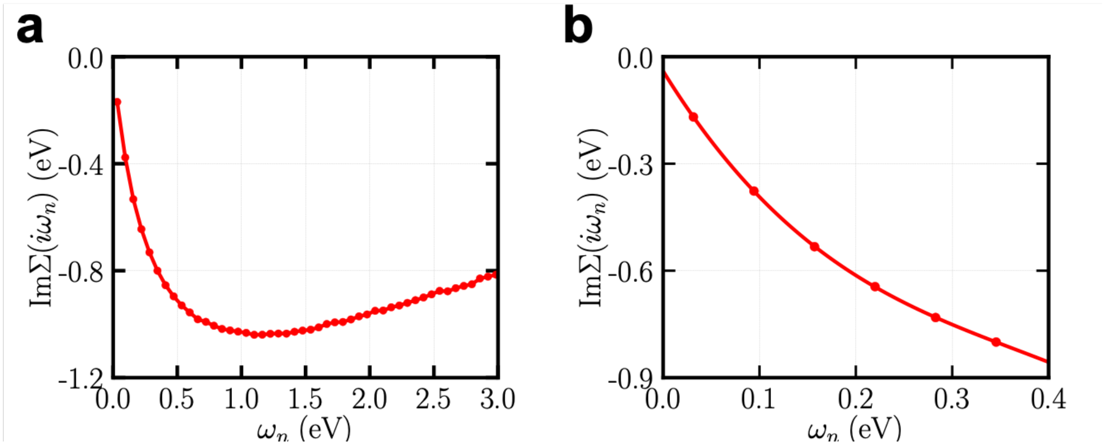


Supplementary Figure 3: (a) The imaginary part of the self-energy of Ni-$d_{x^2-y^2}$ orbital calculated using the low-energy effective model Eq. (1) in the main text with $U_d = 3.7$ eV. (b) The imaginary part of the self-energy of Ni-$d_{x^2-y^2}$ orbital at the first six Matsubara frequencies, calculated using the low-energy effective model with $U_d = 3.7$ eV. The symbols are from DMFT calculations and the curves are from a fourth-order polynomial fitting. The effective mass can be extracted by: $\frac{m^*}{m} = Z^{-1} \simeq 1 - \frac{d\mathrm{Im}\Sigma(i\omega_n)}{d\omega_n}\Big|_{\omega_n\to 0}$.

## Supplementary Note 5. POSSIBLE STRUCTURAL DISTORTIONS IN THE CO STATE

In this section, we study possible structural distortions in the $\mathbf{q} = \left(\frac{1}{3}, 0, 0\right)$ CO state. In the main text, we use the DFT-optimized crystal structure of $NdNiO_2$, which is in good agreement with the experiment. For the PM and AFM states, there are no internal atomic degrees of freedom because all the Ni atoms have the same charges. In the $\mathbf{q} = \left(\frac{1}{3}, 0, 0\right)$ CO state, since there is a charge transfer from Ni2-$d$ to Nd-$d$/interstitial-$s$ orbitals, Ni2 site is inequivalent to Ni1/Ni3 sites. Due to the charge depletion, the two oxygen atoms closest to the Ni2 atom may approach the Ni2 atom, as shown in panel (a) of Fig. 4.

To find the displacements of those two oxygen atoms, we still use the low-energy effective model (Eq. (1) in the main text) with $U_d = 3.7$ eV, which enables us to calculate accurate total energy as a function of oxygen movement $\delta$. We note that while the effective model does not include O-$p$ states explicitly, the effect of the oxygen movement can be modelled by increasing the Ni2 onsite energy. The new Ni2 onsite energy is determined by fitting to the DFT band structure of "distorted" $NdNiO_2$. The total energy as a function of oxygen movement $\delta$ is shown in panel (b) of Fig. 4. We find that an optimal $\delta$ of 0.047 Å does decrease the total energy by about 5 meV/f.u., compared to the DFT-optimized structure. However, such an oxygen movement is minute and thus it is challenging to directly observe this lattice distortion in experiment. The energy gain from the lattice distortion is also small. By comparison, the energy gain in the CO state from the charge transfer is about 50 meV/f.u. (see Fig. 3(b) in the main text). We note that taking into account the structural distortions will further lower the total energy of the CO state, which does not affect our main conclusion that above a critical $U_d$, the CO state can be stabilized over the PM and AFM states.

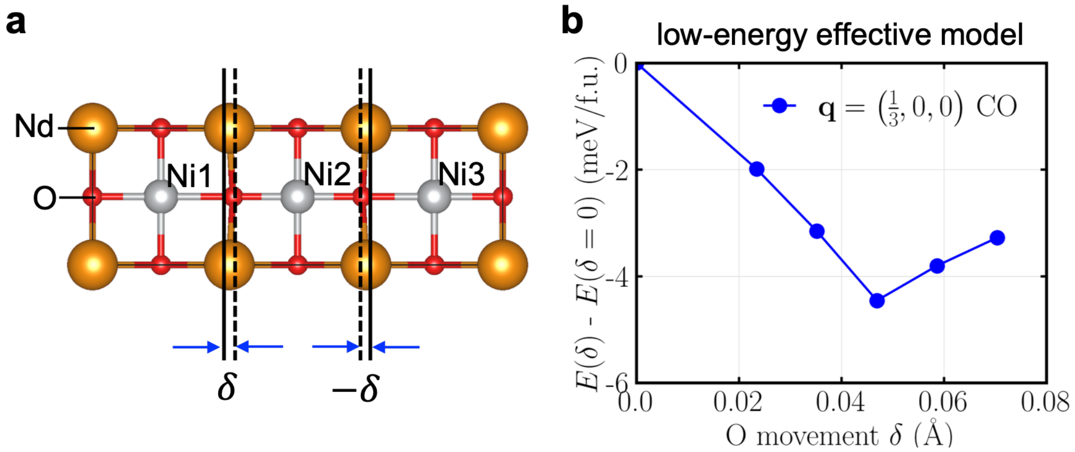


Supplementary Figure 4: (a) Possible structural distortions of $\mathbf{q} = (\frac{1}{3}, 0, 0)$ CO state, in which the two oxygen atoms closest to the Ni2 atom approach the Ni2 atom. Their position movements are denoted by $\delta$ and $-\delta$. (b) The total energy of $\mathbf{q} = (\frac{1}{3}, 0, 0)$ CO state as a function of oxygen position movement $\delta$, calculated using the low-energy effective model with $U_d = 3.7$ eV.

## Supplementary Note 6. COMPARISON OF THE PM AND CO SPECTRAL FUNCTIONS USING THE LOW-ENERGY EFFECTIVE MODEL

In this section, we calculated the PM and CO spectral function using the low-energy effective model (Eq. (1) in the main text) with $U_d = 4$ eV. The results are shown in Fig. 5. Panel (a) is the PM state and panel (b) is the CO state. While the spectral functions calculated with $U_d = 4$ eV are qualitatively similar to those calculated with $U_d = 3.7$ eV, the quasi-particle peak is more strongly suppressed in the CO state as $U_d$ gets larger.

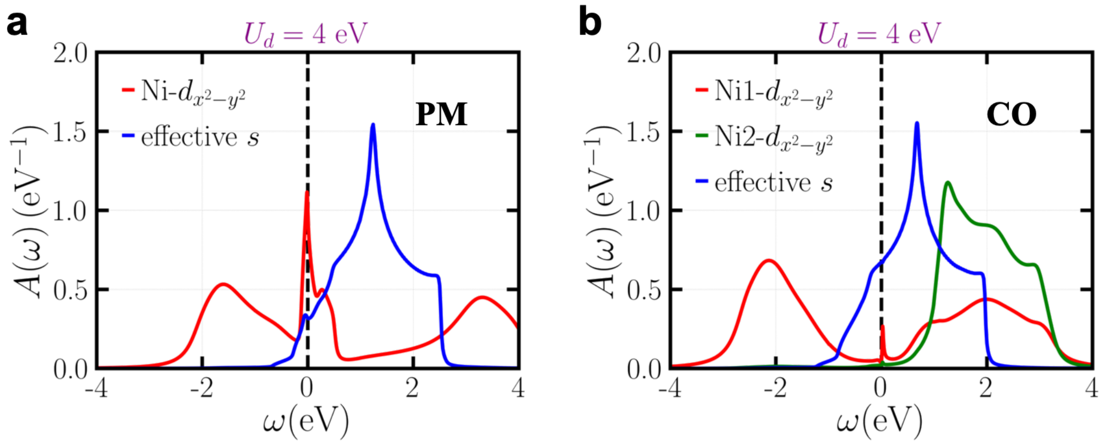


Supplementary Figure 5: Comparison of the orbital-resolved spectral function of the low-energy effective model: (a) the PM state and (b) the CO state. The calculations are performed with $U_d = 4$ eV.

## Supplementary Note 7. TOTAL ENERGY AS A FUNCTION OF $E_{ds}$ CALCULATED WITH $U_d = 4$ EV

In this section, we calculated the total energy of the PM, AFM and CO states as a function of $E_{ds}$ using the low-energy effective model (Eq. (1) in the main text) with $U_d = 4$ eV. We show the results in Fig. 6 and find that they are very similar to those calculated with $U_d = 3.7$ eV (Fig. 5(b) in the main text).

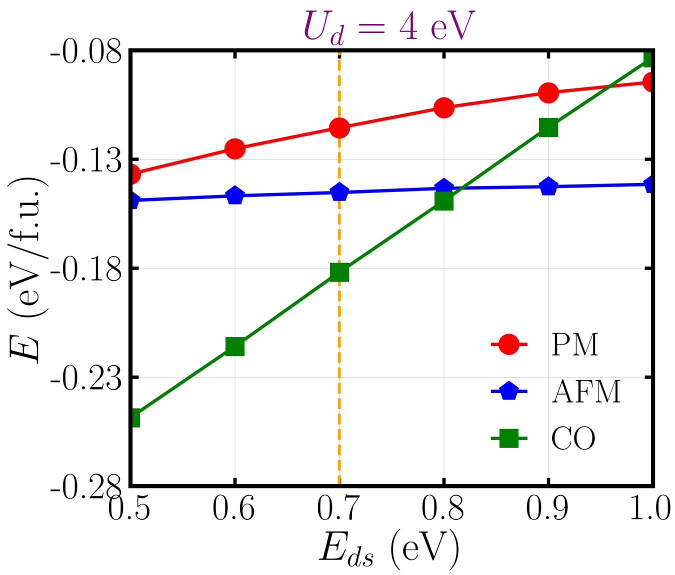


Supplementary Figure 6: The total energy (up to a constant) of the PM (red), the AFM (blue) and the CO (green) states as a function of $E_{ds}$, calculated using the effective model with $U_d = 4$ eV. The orange dashed line highlights $E_{ds} = 0.7$ eV, which is obtained from fitting to the DFT band structure. The error bar is smaller than the symbol size.

## Supplementary Note 8. COMPARISON OF COMPETING STATES USING THE LOW-ENERGY EFFECTIVE MODEL

In this section, we use the low-energy effective model to compare the total energy of the following competing states: uniform paramagnetic state (PM), checkerboard antiferromagnetic state (AFM), $\mathbf{q} = \left(\frac{1}{N}, 0, 0\right)$ charge ordered state (CO) with $N = 2, 3, 4, 5$ (labelled as CO-I to CO-IV) and a different CO state (labelled as CO-V). We find that with the DFT-fitted energy dispersion, for a reasonable range of $U_d$, $\mathbf{q} = \left(\frac{1}{3}, 0, 0\right)$ CO is the most energetically favorable one among all the states considered.

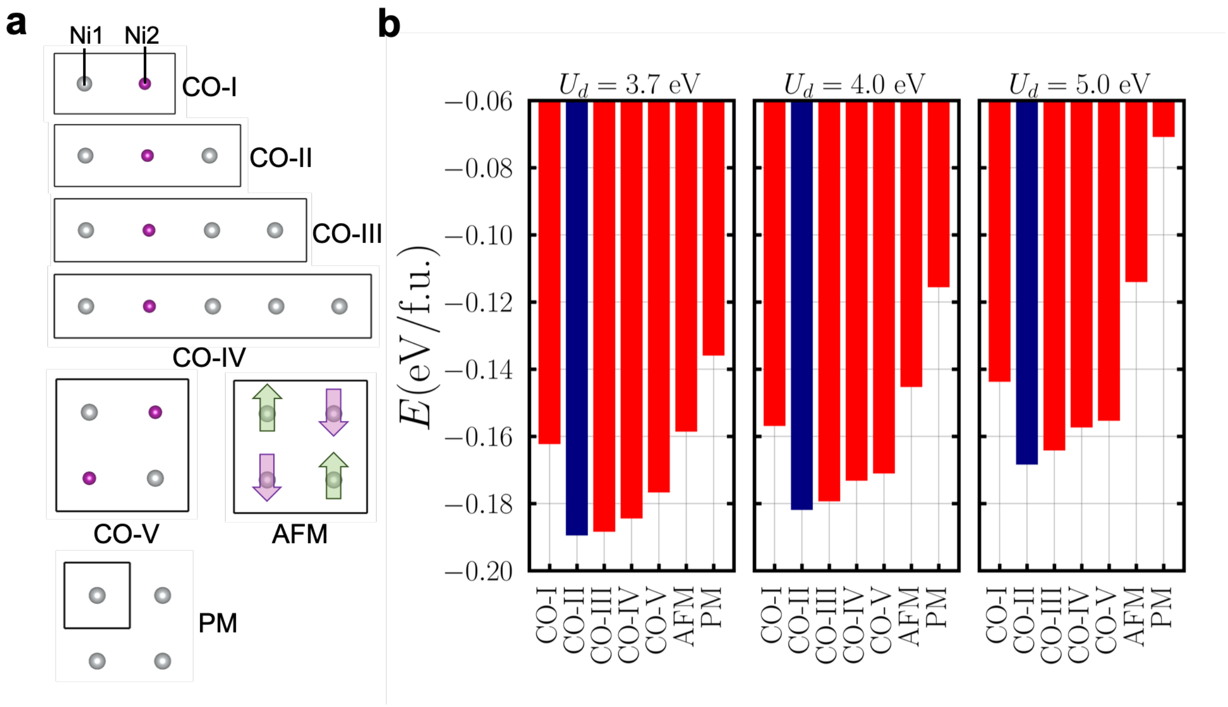


Supplementary Figure 7: (a) Schematic of various competing states. The black box is the simulation cell. We study five different charge ordered (CO) states (labelled as CO-I to CO-V), a checkerboard antiferromagnetic state (AFM) and a uniform paramagnetic state (PM). In the CO state, the purple ball highlights the $Ni^{2+}$ site where the charge transfer occurs and a hole is localized. (b) Comparison of total energy of various competing states calculated by the low-energy effective model. For a reasonable range of $U_d$, the most energetically favorable state is $\mathbf{q} = \left(\frac{1}{3}, 0, 0\right)$ charge ordered state (i.e. CO-II), which is highlighted in dark blue.

[1] P. Hansmann, X. Yang, A. Toschi, G. Khaliullin, O. K. Andersen, and K. Held, Phys. Rev. Lett. **103**, 016401 (2009).

[2] P. Werner, E. Gull, and A. J. Millis, Phys. Rev. B **79**, 115119 (2009).

[3] F. Lechermann, Phys. Rev. X **10**, 041002 (2020).

[4] F. Lechermann, Phys. Rev. B **101**, 081110 (2020).

[5] K. Haule, T. Birol, and G. Kotliar, Phys. Rev. B **90**, 075136 (2014).

[6] K. Haule and T. Birol, Phys. Rev. Lett. **115**, 256402 (2015).

[7] M. T. Czyżyk and G. A. Sawatzky, Phys. Rev. B **49**, 14211 (1994).

[8] I. Leonov, S. L. Skornyakov, and S. Y. Savrasov, Phys. Rev. B **101**, 241108 (2020).

[9] Y. Gu, S. Zhu, X. Wang, J. Hu, and H. Chen, Commun. Phys. **3**, 84 (2020).

[10] J. Karp, A. Hampel, M. Zingl, A. S. Botana, H. Park, M. R. Norman, and A. J. Millis, Phys. Rev. B **102**, 245130 (2020).

[11] F. Lechermann, Phys. Rev. X **10**, 041002 (2020).

[12] F. Lechermann, Phys. Rev. Materials **5**, 044803 (2021).

[13] L. Si, W. Xiao, J. Kaufmann, J. M. Tomczak, Y. Lu, Z. Zhong, and K. Held, Phys. Rev. Lett. **124**, 166402 (2020).

[14] S. Ryee, H. Yoon, T. J. Kim, M. Y. Jeong, and M. J. Han, Phys. Rev. B **101**, 064513 (2020).

[15] Z. Liu, C. Xu, C. Cao, W. Zhu, Z. F. Wang, and J. Yang, Phys. Rev. B **103**, 045103 (2021).

[16] Y. Wang, C.-J. Kang, H. Miao, and G. Kotliar, Phys. Rev. B **102**, 161118 (2020).

[17] N. Marzari, A. A. Mostofi, J. R. Yates, I. Souza, and D. Vanderbilt, Rev. Mod. Phys. **84**, 1419 (2012).

[18] M. Aichhorn, L. Pourovskii, V. Vildosola, M. Ferrero, O. Parcollet, T. Miyake, A. Georges, and S. Biermann, Phys. Rev. B **80**, 085101 (2009).

[19] H. T. Dang, J. Mravlje, A. Georges, and A. J. Millis, Phys. Rev. B **91**, 195149 (2015).

[20] F. Petocchi, V. Christiansson, F. Nilsson, F. Aryasetiawan, and P. Werner, Phys. Rev. X **10**, 041047 (2020).
